# Fast and accurate medication identification

**DOI:** 10.1038/s41746-019-0086-0

**Published:** 2019-02-28

**Authors:** Natalia Larios Delgado, Naoto Usuyama, Amanda K. Hall, Rebecca J. Hazen, Max Ma, Siva Sahu, Jessica Lundin

**Affiliations:** Microsoft Research, One Microsoft Way, Redmond, WA 98052 USA

**Keywords:** Health services, Occupational health, Computer science, Software

## Abstract

Much of the AI work in healthcare is focused around disease prediction in clinical settings, which is an important application that has yet to deliver in earnest. However, there are other fundamental aspects like helping patients and care teams interact and communicate in efficient and meaningful ways, which could deliver quadruple-aim improvements. After heart disease and cancer, preventable medical errors are the third leading cause of death in the United States. The largest subset of medical errors is medication error. Providing the right treatment plan for patients includes knowledge about their current medications and drug allergies, an often challenging task. The widespread growth of prescribing and consuming medications has increased the need for applications that support medication reconciliation. We show a deep-learning application that can help reduce avoidable errors with their attendant risk, i.e., correctly identifying prescription medication, which is currently a tedious and error-prone task. We demonstrate prescription-pill identification from mobile images in the NIH NLM Pill Image Recognition Challenge dataset. Our application recognizes the correct pill within the top-5 results at 94% accuracy, which compares favorably to the original competition winner at 83.3% for top-5 under comparable, though not identical configurations. The Institute of Medicine claims that better use of information technology can be an important step in reducing medication errors. Therefore, we believe that a more immediate impact of AI in healthcare will occur with a seamless integration of AI into clinical workflows, readily addressing the quadruple aim of healthcare.

## Introduction

The third most common cause of death is not disease, but medical error, with 250–400 k or more mortalities per year.^1–5^ The epidemic of medical error gained attention in reports from the Institute of Medicine,^3,6^ which found that the most common type of preventable medical error is medication error, which results in over 1.5-m injuries and over $3b in complication costs alone. The Institute of Medicine’s 2006 report further provides guidelines on reducing the high frequency and unacceptable cost of medication error, including greater use of information technology, which could be implemented at each stage from prescribing and dispensing through to monitoring the patient's response. Technology solutions have been applied to drug reference information, drug–drug interactions, drug allergies, and threshold warnings for high doses. Despite the common occurrence, there is little research funding in medical error, particularly when compared with other leading causes of death, such as heart disease and cancer. However, there are clear cost benefits; computerized medication systems have the potential to reduce errors by 84% and save hospitals over $500 k/year in direct costs.^7^

In 2015, the triple aim:^8^ patient experience, outcome, and cost, became the quadruple aim to include care-team experience.^1^ Personalized medicine squarely addresses the outcome, with pioneering advances in research;^9,10^ however, personalized medicine is nascent years or generations from generalizing in earnest in clinical settings.^11–14^ For example, we show how deep learning can more immediately address the quadruple aim by providing tools that improve task efficiency and seamlessly fit into clinical workflows. This improves the patient and care-team experience while improving quality and cost, and furthermore, as demonstrated here, these technologies already generalize beyond specific settings or use cases, here on real-world, mobile-generated images.

To provide appropriate healthcare and avoid medication errors, it is paramount to know which medications a patient is taking.^15^ Discrepancies in medication are common, over half of patients in one study at the time of hospital admission, with 39% capable of causing injury, and the most common type of discrepancy is errors of omission, leaving out medications a patient is taking.^16^ It is frequently a challenge for consumers to identify pills when pills are transfered to different containers, combined to a single container for convenience, or portioned into day-of-week pillboxes to simplify medication management. While generally well intended, when patients separate medications from their original bottles or packaging, this presents a challenge to their healthcare teams. Pharmacies often host brown bag consultations,^17^ where patients are encouraged to bring in their unknown pills in brown-paper bags for pharmacists to identify. A reference search by hand-entering physical characteristics (color, shape, and imprint) of over 10,000 FDA-approved medications^18^ is a slow, tedious, and error-prone process that requires dexterity to handle small pills, vision to read small writing, and some degree of health literacy.

Breakthroughs in prescription medication are among the reasons we live longer. For example, HIV is today a chronic disease,^19^ while it was a fatal diagnosis in the 1980s. Meanwhile, there is an increasing number of medications, both branded and generic on the market as the number of FDA-approved drugs continues to increase.^20^ At the same time, prescription medication usage is increasing; over 4 billion prescriptions were filled in 2017.^21^ In a given week, four out of five people take prescription, over-the-counter, and supplementary medications, and one-third of people will take five or more.^3^ Medication usage is increasing across age groups, particularly among the elderly. Of people aged 65 and above, nine of ten were on prescription medications within the last 30 days.^22,23^ In the same age group, the consumption of multiple medications is common. Despite high rates of medication usage, as a population, we are getting sicker. In 2012, about half of the Americans had at least one chronic condition, and one in four had multiple chronic conditions,^24^ a growing population that frequently requires multiple medications. Thus, increasing medication usage across the population puts additional responsibility on patients to take medications as prescribed and making medication errors more likely outside hospital settings. Between 2003 and 2007, there was a 44% increase in calls to poison control centers^25^ with most of the increased calls relating to pill identification. From 2000 through 2012, Poison Control Centers in the United States received data on 67,603 exposures related to unintentional therapeutic pharmaceutical errors that occurred outside of healthcare facilities that resulted in serious medical outcomes. The overall average rate of these medication errors was 1.73 per 100,000 population, resulting in 414 deaths, and there was an increase from 3065 to 6855 during the 13-year study period.^26^ In 2012, nearly 300 k people called poison control regarding medication error, 16% for taking the wrong medication.^27^

Due to the US intellectual-property law, drug manufacturers own the physical characteristics of the medication, including size, shape, color, texture, and aroma. The motivation behind this legislation was to reduce opportunities for counterfeit medications. The unintended consequence today is that the patient may experience wide ranges in pill appearance when refilling a medication due to changes in generic brands. Currently, generic medications are 70% of the US market, where some drugs (e.g., fluoxetine) can have over 10 variations of generics. It is easy to imagine how the plethora of drugs and drug formulations can create confusion that can negatively impact adherence, medication error, and complexity as part of a medication regimen.^28^

*Automated pill recognition*. Recognizing the need to push for innovation around automated pill identification, the NLM hosted a Pill Image Recognition Challenge^2^ in 2016. Although the challenge deadline has already passed, the dataset provided offers valuable consumer images, and the winning model offers a baseline for improving upon pill recognition and identification task. The dataset contains two types of images, one type imitating pictures taken by mobile users and the other consisting of images submitted by pharmaceutical companies to the NIH, which are respectively referred as consumer and reference images. The winner of NLM’s pill challenge^29^ used an approach combining pill localization based on simple gradients and morphology operations for reference images and support vector machine-based pill detection operating over regions represented by a histogram of oriented gradients^30^ for consumer ones, with pill identification based on a modified deep-ranking^31^ approach. The paper authored by the challenge winners focuses to a large degree in the creation of lighter mobile-ready versions of their base model. Following the NLM challenge, two other deep-learning approaches were proposed. Wang et al.^32^ proposed a pill recognition system using GoogLeNet Inception Network^33^ with Canny edge detection^34^ for pill localization. Wong et al.^35^ proposed an AlexNet^36^-based approach. Remarkably, the authors created their own dataset, consisting of 400 “commonly-used tablets and capsules”. Though the orientation, position, and lighting of these tablets are much more controlled than those in the larger NLM dataset. This is also a classification-based approach, as the survey of models that we present in the Methods section.

Apart from the deep-learning-based approaches, various feature engineering methods have been proposed. For capturing shape features, Lee et al.^37^ proposed Hu moment^38^ and grid intensity methods. Hu moment was also proposed by Cunha et al.^39^ because of its rotation-invariant nature. Caban et al.^40^ proposed another rotation-invariant feature, which adds up the distances from the centroid to the contour of the pill. Focusing on imprint representation, Yu et al.^41^ proposed Modified Stroke Width Transform to obtain imprint stroke features. For color characteristics, Caban et al.^40^ proposed a HSV color histogram-based method. A HSV color model was also employed in the studies by Cunha et al.^39^ and Yu et al.^42^ to eliminate the disturbance of luminance. With the hand-crafted features,^40^ Wong et al.^35^ reported 98.55% top-5 accuracy of 400 pills using Random Forest.^43^ The evaluation is based on the corrected images using their color marker.

Our research revisiting automated recognition of pills is motivated by current trends in healthcare relating to the increasing administration of prescription medications in pill form; the emphasis is on medication reconciliation as a quality and safety initiative (the Centers for Medicare and Medicaid Services, the Institute for Healthcare Improvement, and The Joint Commission's National Patient Safety Goals), and the great advances in image classification in the last few years, thanks to new developments in deep learning. We employ the data afforded by the Pill Recognition Challenge to perform a series of experiments that create and evaluate image classifiers powered by different deep Convolutional Neural Network (CNN) models in the task of recognizing pills from images. The results of these experiments allow us to select the best model and parameter configuration to create a proof-of-concept pill identification service that already provides high-certainty predictions on the set of pills that it was created. Our results, when considered for a real-world pill identification implementation, dramatically outperform those of the deep-ranking-based approach of the challenge winner. Thus, the experimetal results presented below made us decide to continue with a traditional multi-class approach, as we aim to increase the number of supported drug codes. Even so, we are fully aware that at some point, we may need to consider approaches based on extreme multi-label prediction or ranking to support a much larger number of codes (currently several tens of thousands).

## Results

### Classification accuracy

The following recognition results abide to an experimental setup that finds an optimal hyper-parameter configuration to learn each model, which also provides a realistic and fair evaluation. This is a very important consideration, given our desire to power a pill-identification service with the resulting model. Details about the dataset we employ and the differences between reference and consumer pill images can be found in the Methods section. Our protocol is comprised of using a hold-out set formed by 20% of the consumer images set combined with a hyper-parameter search consisting of a fourfold cross-validation (CV) on different parameter value combinations for each CNN model-configuration setup. The CV and final model learning are carried out with the remaining 80% consumer images and all the reference ones.

Table 1 contains the top-1 and top-5 accuracy results obtained in the best CV average (CV avg.) in the configuration search for each model and (Evaluation) with the best configuration in the hold-out consumer images. The CV results shown are for the configuration with the highest top-5 CV avg of each model. A final instance is learned with this configuration, which was then applied to the hold-out test to obtain an estimate as close to the performance of a service using any of these models in the real world.

**Table 1:** Summary of model accuracy results on evaluation of consumer images where the correct image is the first result (Top-1), or within the first five results (Top-5)

	Best CV avg.		Evaluation			Model	
	Top-1	Top-5	Top-1	Top-5	Avg precision	Parameters [millions]	Depth
	Accuracy, %		Accuracy, %		All classes, %		
ResNet50^45^	71.72	92.85	77.00	95.3	85.87	26.35	168
MobileNet^53^	71.7	92.00	77.10	94.40	84.29	5.04	88
SqueezeNet^52^	49.05	76.80	56.20	83.20	61.33	2.06	18
InceptionV3^54^	74.42	93.30	76.30	94.80	85.94	26.56	159

The table also includes average precision of the model over all classes along with its total number of parameters to assess its complexity

The Methods section includes details about the CNN models we surveyed and the hyper-parameters value configuration we have to find. We set up a hyper-parameter search space consisting of value pairs from the Cartesian product [0.2,0.4] × [5 × 10^−5^, 1 × 10^−4^, 5 × 10^−^^4^] between predefined sets of drop-out and initial learning-rate values. The configuration of each model with the highest average performance in CV is then used to perform a final model learning process, which is then evaluated on the never-seen hold-out dataset of consumer images.

Inception V3 had the best average top-5 accuracy during CV, closely followed by ResNet50 and surprisingly MobileNet. The two leading models swapped the performance lead with the hold-out dataset. It is also notable that MobileNet displayed performance levels very close to these two top models that have five times the number of parameters. SqueezeNet is the smallest and least complex type of model that we evaluated for this domain, and it clearly showed in its much lower level of performance. Though not displayed in these results, SqueezeNet also had the largest variance in CV accuracy during the hyper-parameter search with one configuration only achieving 34.35% of top-5 accuracy.

As a baseline reference, the average top-1 and top-5 accuracy reported in the MobileDeepPill^29^ are 26.0 ± 1.2% and 53.2 ± 1.9% for the single-CNN version, and 53.1 ± 1.0% and 83.1 ± 0.9% for the multi-CNN one based on a retrieval scheme. These metrics are computed over fivefold CV partitioning by pills. Thus, they are not directly comparable with the numbers in Table 1, since the authors partition image data by pill, thanks to the retrieval-based approach they propose; meanwhile, we only split sets by image. In addition, they use the appearance-based identifiers defined in the challenge, while we directly use NDCs as identifiers, which for some pills, this means combining multiple appearances under the same code, making our task more complex. Still, the MobileDeepPill metrics work as an identification performance baseline when considering which type of approach to apply for a real-world deployment.

### Precision analysis

**Fig. 1:**
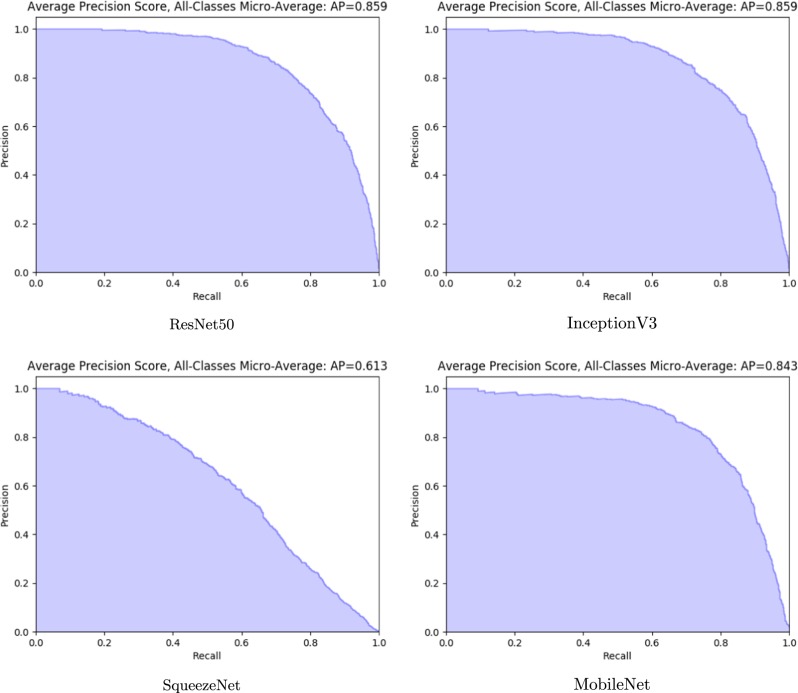
Precision-recall curve plots on a hold-out set comprised from micro-averages of the precision values for each pill class. The plots in clockwise order staring from the upper-left corner are produced by evaluating models learned with the best hyper-parameter configurations for the ResNet50, InceptionV3, MobileNet, and SqueezeNet networks

Figure 1 contains the evaluation precision-recall (PR) curve plots of each model calculated using per-class micro-averages. These plots illustrate well the differences in the overall evaluation performance between models. ResNet50 and Inception V3 have practically identical PR curves and average precision scores. MobileNet has a slightly lower identification performance due to lower precision averages at a low-recall range.

### ResNet50 detailed analysis

We now focus on the ResNet50 model, which displayed the best evaluation performance in our setup, and which we already use in our proof-of-concept implementation.

**Fig. 2:**
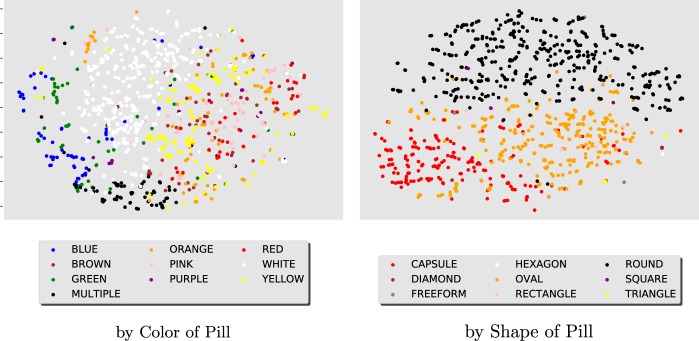
t-SNE visualizations of the high-dimensional outputs obtained by evaluating the selected ResNet50 model on consumer images of the hold-out set. They are obtained from the last layer of the model before the fully connected one. The t-SNE plot on the left colors similarly all dots from images with the same pill color. The right plot colors all dots based on pill shape

We create t-SNE^44^ visualizations (Fig. 2) of the final high-dimensional hidden layer of the ResNet50 model of the hold-out consumer images. By visualizing in 2d the model output, we see that the CNN model detects groupings of similar pill categories of color and shape, without explicitly including these features in the model training.

**Fig. 3:**
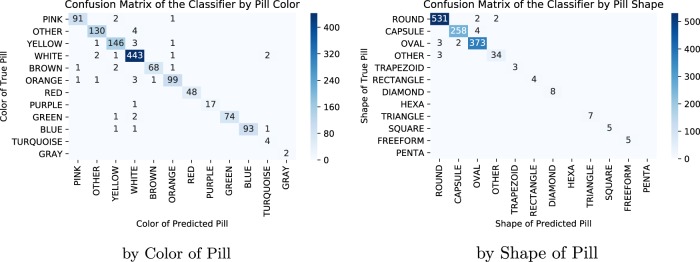
Confusion matrices of ResNet50 predictions grouped by pill characteristic. The columns indicate the predicted-pill characteristic, while the rows the true-pill ones. The left matrix adds by color of the pill prediction and its true color and the right aggregates based on shape. To reduce crowding, zero counts are not shown

We also created confusion matrices for this model’s predictions on the hold-out consumer images. First, we had created a confusion matrix of NDC predictions as it is standard in reporting classification performance, but given the model’s high performance and the experiment’s high pill count, it was hard to make out the details of it. Thus, we include two confusion matrices that group the hold-out images by color and by shape of the predicted-pill NDCs (Fig. 3) that have few images outside the diagonal, since most of the mistakes made by the model are intrashape and color. This is also clearly shown when looking at the pills in which the model obtained its lowest average precision scores.

**Fig. 4:**
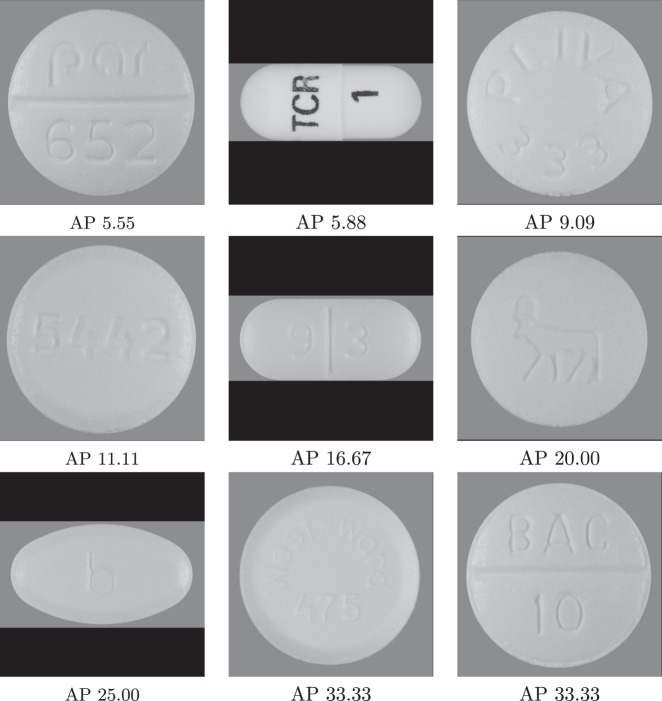
Example images of the lowest average precision [%] pill classes in the ResNet50’s evaluation. The images are in increasing order starting with the lowest-scoring pill class at the top-left corner

Finally, we present the pills that had the lowest average precision in Fig. 4. It is easy to understand the reason why these particular pills are worsening the model performance. In fact, groups of these pills in combination are the hardest for the model since they are easy to confuse. Most of them have an engraved imprint, which is more difficult to read. These pills are a clear example of the intrashape and color mistakes indicated by the confusion matrices above, since they all are either white round tablets or capsules.

## Discussion

The results shown in our survey of the existing CNN technology applied to pill identification demonstrate that recent advances in AI make it feasible to mostly automate this task involving pharmacies, patients, first responders, and care providers. In general, this work serves as an example that one of the initial impacts of AI in healthcare will be to streamline tasks, before getting into supplanting care staff in complex and high-stake tasks, such as diagnosing and prognosticating, if ever. On the other hand, AI-powered tools like ours will allow care teams to focus more on the patient increasing their productivity and deceasing the risk of error. It is through these mechanisms that our system can have an effect in the quadruple aim.

For the first proof-of-concept implementation of our pill recognition service, we went with a Resnet50^45^ model as the final classifier. Both MobileNet and SqueezeNet are simpler deep CNN-based classifiers aimed at running in mobile devices, which provide an interesting contrast with our initial ResNet50 selection. On the other hand, InceptionV3 has a network structure of comparable depth and number of parameters. The results of this evaluation are the basis from which we will select the type of model to power the next iteration of our recognition service. Current results indicate that ResNet50 continues to be a good option, with Inception V3 and surprisingly MobileNet also having comparable performance in this domain.

As future work, expanding the dataset for different camera angles and configurations, and lighting conditions would be required to ensure model performance in practice. The consumer images in the competition dataset are challenging with various lighting conditions; however, they are still relatively controlled with the same layout and the camera angle. Expanding the dataset with more pill types would be also important to support more use cases. We plan to look into extreme classification^46^ and metric- learning^47^ techniques to handle a much larger range of pills. Although our identification approach performed quite well with 924 target pills, giving coverage to the tens of thousands FDA-approved pills will be challenging. In addition, as the pills with low average-precision score indicate (Fig. 4), it is necessary to employ advanced OCR “in the wild” techniques (i.e., from photographs of signs and lettering instead of documents) in order to improve identification accuracy since many of these low-precision pills are all but identical with the exception of their hard-to-read engraved imprint. These areas constitute two rich and interesting avenues for future work.

We believe that current developments in AI make it possible to create systems that will have an immediate impact in multiple pharmacy and first-responder processes throughout the healthcare industry. These AI-powered systems and services have the potential to change for the better how patients and healthcare teams communicate and interact with each other, as well as, how staff and practitioners are empowered by smart tools that help them to be more efficient and accurate.

## Methods

### Data and preprocessing

The Pill Image Recognition Challenge dataset consists of 7000 pill images of 1000 pill types specifically designed for the contest. The images are divided into 2000 reference images and 5000 consumer-quality ones. Reference images have controlled lighting and background, while the consumer-quality ones have variable conditions. Consumer images additionally vary in focus and device type. They imitate the pictures taken by users that would be sent to an automated pill-recognition system.

#### Labels based on National Drug Codes

The National Drug Code (NDC) is a unique 10-or-11-digit, 3-segment number. It functions as a universal product identifier for human drugs in the United States. Our implementation goals for recognizing pills require providing medication information based on the predicted identity of the pills. Thus, we decided to use the NDC label and product codes of the pills available as part of the challenge dataset identifiers instead of the original labels provided by the NLM dataset creators. Some pills in the dataset were put into different classes, given that they are versions of the same NDC with different appearances. Thus, as we changed into NDC-based labels, some of these pills merged into a single group producing a 924-class dataset for our experiments.

### Identification system overview

Pills are identified employing two deep-learning models in series. First, we perform image segmentation isolating the pill from the background with a blob-detection CNN and define a bounding box to crop a smaller image that centers on the pill. Second, we employ a deep-learning-based classifier to return a ranked list of drug codes based on matched likelihood by the pill in the cropped image output by the initial stage. Figure 6 shows the system architecture overview. In the following sections, we detail the learning setup for the models of each stage. Next, we present the results of the experiment we carried out, comparing multiple CNN models to select which one we will use in our pill identifier implementation. We include additional results deepening the performance evaluation of the selected model.

**Fig. 5:**
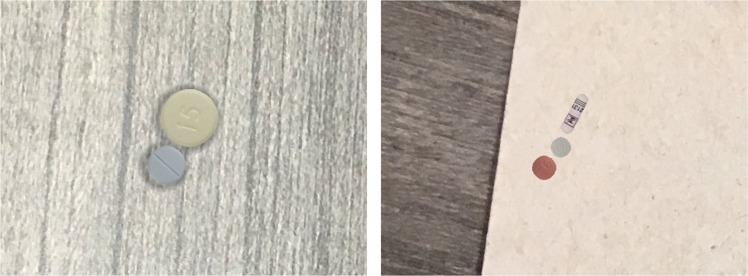
Example synthetic images for the segmentation model training. These images are randomly generated with multiple backgrounds, pills types, and lighting conditions to augment the training set for the segmentation model

**Fig. 6:**
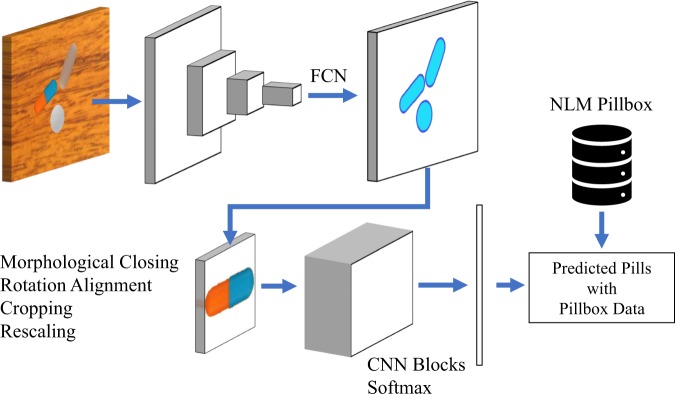
An overview of our pill detection and classification approach. A fully convolutional network (FCN) is used to detect the pill blobs, followed by some standardization steps, including the rotation alignment and scaling. The cropped single-pill images are passed to the CNN blocks to generate the feature vectors and the probabilities

### Pill localization

Our pill localization approach consists of a blob-detection neural network and morphological post processing. For the blob detector, we trained a fully convolutional network (FCN),^48^ which is a pixel-segmentation algorithm. Since the NIH competition dataset does not provide the pill localization data, e.g., bounding boxes, we generated synthetic images and trained FCN only with the synthetic images. The segmentation performance was evaluated indirectly through its effect on the overall classification accuracy as a proxy.

#### Synthetic training images

We generated 100,000 synthetic images of size 480 × 640 for the segmentation model training. From the NIH reference pill images, the background region with RGB color (117 ± 1, 117 ± 1, 117 ± 1) was cropped. We collected various background images, including papers, desks, carpets, and metal textures. They were taken mainly in indoor office environments, but different lighting sources were used. The pill images were superimposed on the background images with the following parameters:

- 1–5 pills per image
- 360° rotation
- 20–50 px pill size
- 0–20 px drop shadow

Example synthetic images are shown in Fig. 5. During the model training runtime, various image augmentations were applied, including contrast and brightness adjustment, Gaussian-blur, and affine and perspective transformation to increase variety in our training set.

#### Blob Detection Neural Network

Our blob detector takes an input image of 480 × 640 × 3 and generates a predicted mask of 480 × 640 × 1. The network architecture differs from the original FCN model in terms of the input image size and the number of output classes. However, we can still leverage transfer learning using the ImageNet^49^ pre-trained weights because FCN does not have any fully connected layers at the top. We replaced the final softmax layer with the sigmoid layer as the output is only two classes (pill and background). To improve the segmentation accuracy around the pill boundaries, we increased the loss weight by two times around the pill boundaries, which is a similar technique used in U-Net.^50^ An Adam optimizer^51^ was used with the initial learning rate of 1e−3. The learning rate was decreased by multiplying 0.2 whenever a plateau in the validation loss was detected. The network was trained until the performance stopped improving. Given the predicted masks, we apply a closing morphology operation of 10 px kernel to remove small blobs. For the consumer images in the dataset, the detected blobs were dilated with 10 px kernel to avoid cutoff for difficult cases. The connected components in the masks are extracted and passed to the next pipeline along with the bounding boxes.

### Identification model input

After the pills in an image have been found by the segmentation stage, a tight bounding box is created around each pill to crop an image and scale it into a 224 × 224-pixel resolution. Note that we do not segment the contour using the predicted masks to avoid cutoff but to also keep the lighting context information in the background texture. The pills are placed in a centered position and padding is inserted in order to maintain the proportion of each pill while creating a square input image.

### CNN models for identification

Deep CNNs have been proven successful in multiple tasks identifying object categories in pictures taken with realistic conditions of varying illumination, focus, and perspective. For this reason, we decided to employ a classification framework instead of a similarity-based one, as it was encouraged in the experimental setup of the pill recognition challenge where we got the data for these experiments.

The main goal of this work is to reliably identify pills from images under any imaging condition in order to provide accurate medication information. As such, we chose classifiers that optimize obtaining the highest accuracy rate with robustness to these challenges when users freely take pictures.

To obtain the models for pill classification, we fine-tune starting from model weights pre-trained on ImageNet. In this paper, we provide a comparison between results of applying ResNet50, SqueezeNet,^52^ MobileNet,^53^ and InceptionV3^54^ models as the final identifier. We modify the original structure by removing the classification layers at the top, e.g., until the last average pooling for ResNet50. We connect the output of this layer with two sequential blocks, each consisting of batch-normalization, drop-out, and dense layers. The rate of these new drop-out layers is left as a hyper-parameter of each model training process. We use an Adam optimizer^51^ whose initial learning rate constitutes the second hyper-parameter whose value we will find by search. The rate is decreased to 0.2 every time learning stops after an epoch, as indicated by the validation loss not decreasing. The weights for each model are all learned with the same fine-tuning strategy starting from the pre-trained weights.

### Pill-identification service implementation

The implementation of our pill identification service is composed of two web-based APIs that handle segmentation first and then identification. The APIs are hosted in separate Azure VMs with a Python implementation based on the Flask framework, and using models with a TensorFlow^55^ back end. The segmentation service locates pills in the image it receives as a parameter. It responds with a list of bounding-box and confidence-score pairs for all the pills it has found. The client is then responsible for cropping the source image based on the received bounding boxes to obtain pill-centered images. Requests to the identification service also require an image as a parameter. These are expected to be generated from the first API, since the identification model is trained and validated on images generated with it. In particular, the second API works on the assumption that there is only one pill per image, roughly centered and covering a big portion of its area. The service response consists of a ranked list of the top-5 pill identity predictions of the model based on confidence. These predictions consist of an NDC and a confidence-score pair for each of the pill image possible medications. The ordered list of NDCs are combined with related information from NLM Pillbox^18^ for the client-side convenience, although it is optional. In terms of the runtime speed, API response time from a 4G mobile network is estimated to be 0.14 s and 0.05 s for the detection API and the identification API, respectively. API end-to-end performance was measured using simulated 4G (upload 3.5 Mb/s, download 4.0 Mb/s, 20 round-trip time) and 3G (upload 500 kb/s, download 750 kb/s, 100 RTT) mobile networks. An Azure VM with Nvidia Tesla V100 GPU and Intel Xeon CPU E5-2690 v4 (2.60 GHz) was used for running the service. The details are shown in Table 2.

**Table 2:** Runtime performance of our pill identification service

	Model		API end-to-end (4G)		API end-to-end (3G)	
	GPU	CPU	GPU	CPU	GPU	CPU
Detection API	0.02 s	1.04 s	0.14 s	1.21 s	0.70 s	1.67 s
Identification API	0.02 s	0.22 s	0.05 s	0.28 s	0.26 s	0.42 s

API end-to-end performance was measured using a throttled network to simulate 3G and 4G mobile networks

Here, we presented a prescription-pill identification method based on a fully convolutional network (FCN) employed as a blob detector. One of the benefits of using a fully convolutional network is that the background textures can be removed from pill images using the predicted segmentation masks. Another advantage is that our approach can locate multiple pills in an input image. This method is accurate, scalable, and rapidly deployed as an API, and has direct applicability in medication reconciliation in addition to other use cases not limited to administration of medications, adherence, and counterfeit detection, the latter estimated to be a $75b challenge.^56^ While we believe that training and testing a model on mobile images will likely result in a method that generalizes well, further investigation is needed to study the efficacy of a similar technology in a pharmacy, ER, or other healthcare (or clinical) setting. We demonstrate with this use case the application of deep learning to empower patients and care teams with tools that streamline tasks and directly impact all four points of the quadruple aim:^1^ improving the patient experience, outcome, cost, and care-team experience.

### Code availability

The experiments and data analysis were carried out using Python 3.5 with the following openly available libraries: tensorflow 1.3.0, keras 2.0.8, numpy 1.15.0, opencv 3.4.2, and sklearn 0.19.0. The code to fine-tune the models was based on the Keras neural network library available at https://keras.io.^57^ The tuning code is proprietary and might be available upon request and under a nondisclosure agreement.

### Reporting summary

Further information on experimental design is available in the Nature Research Reporting Summary linked to this article.

## Supplementary information

Reporting Summary

## Data Availability

The pill images employed for these experiments is an open dataset available for download at https://pir.nlm.nih.gov/challenge/^2^ hosted by the NLM. The starting models and the Keras neural network library used for all the code in this work are available at https://keras.io.^57^

## Supplementary information

**Supplementary Information** accompanies the paper on the *npj Digital Medicine* website (10.1038/s41746-019-0086-0).
